# The impact of maternal hepatitis B virus infection on IVF/ICSI-assisted pregnancy outcomes: a propensity score-matched cohort study

**DOI:** 10.3389/fmed.2026.1925066

**Published:** 2026-09-03

**Authors:** Wenwen Jiang, Suqin Zhu, Rongshan Li, Yan Sun, Xiaojing Chen, Beihong Zheng

**Affiliations:** 1 Center for Reproductive Medicine, Fujian Maternity and Child Health Hospital, Affiliated Hospital of Fujian Medical University, Fuzhou, China; 2 Fujian Maternal-Fetal Clinical Medicine Research Center, Fuzhou, China; 3 Fujian Key Laboratory of Prenatal Diagnosis and Birth Defect, Fuzhou, China

**Keywords:** hepatitis b virus, *in vitro* fertilization, intracytoplasmic sperm injection, pregnancy outcome, preterm birth, propensity score matching

## Abstract

**Background:**

Hepatitis B virus (HBV) infection is highly prevalent among women of reproductive age in China, yet whether maternal HBV carrier status affects the outcomes of assisted reproduction remains unclear. Published studies have yielded conflicting results, most were underpowered or inadequately controlled for confounders, and few have tracked outcomes beyond early pregnancy. This study used propensity score matching to evaluate the impact of maternal HBV infection on laboratory, clinical pregnancy, obstetric, and neonatal outcomes in women undergoing their first fresh IVF/ICSI embryo transfer cycle.

**Methods:**

This retrospective study analyzed clinical data of 3,455 cycles involving couples who underwent fresh IVF/ICSI embryo transfer for the first time at the Reproductive Center of Fujian Maternal and Child Health Hospital from January 2018 to December 2020. Patients were divided into the HBV group (*n* = 811) and non-HBV group (*n* = 2,644). Propensity score matching was used to perform 1:1 nearest-neighbor matching on baseline covariates, yielding 810 pairs with balanced covariates.

**Results:**

After propensity score matching, baseline characteristics were well balanced. No significant differences were observed in oocyte maturation rate (52.84% vs. 52.38%), normal fertilization rate (81.73% vs. 82.01%), high-quality embryo rate (57.82% vs. 58.66%), blastocyst formation rate (63.49% vs. 64.45%), implantation rate (30.99% vs. 32.76%), clinical pregnancy rate (44.44% vs. 45.43%), multiple pregnancy rate, miscarriage rate (12.78% vs. 9.24%), stillbirth rate (1.39% vs. 1.09%), ectopic pregnancy rate, or live birth rate (32.59% vs. 32.96%) (all *P* > 0.05). Rates of gestational hypertension, gestational diabetes, premature rupture of membranes, cesarean delivery, and neonatal outcomes were also comparable. However, the preterm birth rate was higher in the HBV group both before (15.73% vs. 11.10%, *P* = 0.041) and after matching (15.73% vs. 7.78%, *P* = 0.004).

**Conclusions:**

In this propensity score-matched cohort, maternal HBV infection was not associated with impaired laboratory, clinical pregnancy, or live birth outcomes following IVF/ICSI with fresh embryo transfer. Apart from an increased preterm birth rate, no significant differences were observed in obstetric complications or neonatal outcomes. The association with preterm birth warrants further investigation in prospective studies incorporating detailed virologic data.

## 1 Introduction

Hepatitis B virus (HBV) infection remains a major global public health concern, with an estimated 254 million people living with chronic infection worldwide (1). China is among the regions with the highest HBV prevalence, accounting for nearly one-third of chronic HBV carriers globally, and mother-to-child transmission contributes to 30%–50% of these chronic infections (2, 3). As a result, a substantial proportion of women of reproductive age in China are HBV carriers, and approximately 1.5 million HBV-infected women give birth each year. For these women, fertility care—including counseling on assisted reproductive technology (ART)—has become an increasingly common clinical scenario.

Mechanistic studies have shown that HBV DNA can integrate into human sperm chromosomes, while HBV-related viral material has also been detected in ovarian and follicular compartments of infected women (4–6). These findings have raised concerns that HBV infection may impair gametogenesis, fertilization, and early embryonic development, and may also affect placentation and obstetric outcomes through chronic immune activation or local inflammatory responses (7–10). For couples undergoing *in vitro* fertilization or intracytoplasmic sperm injection (IVF/ICSI), such concerns translate into practical clinical questions: does maternal HBV infection compromise the chance of a healthy live birth, and does it increase the risk of adverse obstetric or neonatal events?

However, the existing evidence addressing these questions remains limited and conflicting. Some studies have suggested that HBV infection may be associated with impaired sperm quality, fertilization, or early embryonic development (9, 11), whereas other studies have found no significant adverse effects on clinical ART outcomes. For example, a large cohort study by Wang et al. reported no significant adverse effect of maternal HBV infection on IVF outcomes (12), and Hu et al. found that the presence of HBV in oocytes and embryos was not associated with pregnancy outcomes following fresh or frozen embryo transfer (13). In contrast, Lam et al. reported higher pregnancy and implantation rates among HBV-seropositive couples undergoing IVF and embryo transfer (14). This uncertainty is also reflected in the meta-analytic evidence: a meta-analysis suggested that HBV infection may adversely affect some IVF-related outcomes, although substantial heterogeneity among the included studies limited the strength of its conclusions (15). Collectively, these discrepant findings underscore the lack of consensus regarding the reproductive implications of HBV infection in the context of ART. Importantly, most prior investigations were limited by small sample sizes, inadequate adjustment for confounders such as ovarian reserve markers, stimulation protocols, and insemination methods, and a narrow focus on early pregnancy endpoints. Few studies have simultaneously reported laboratory, clinical pregnancy, obstetric, and neonatal outcomes within a single cohort, leaving an incomplete picture of the overall impact of maternal HBV on ART-assisted pregnancies.

To address these gaps, we conducted a retrospective propensity score-matched cohort study to comprehensively evaluate outcomes spanning oocyte retrieval, fertilization, embryo quality, clinical pregnancy, obstetric complications, and neonatal health in HBV-infected vs. non-infected women undergoing their first fresh IVF/ICSI embryo transfer cycle.

## 2 Materials and methods

### 2.1 Patients

**Figure 1:**
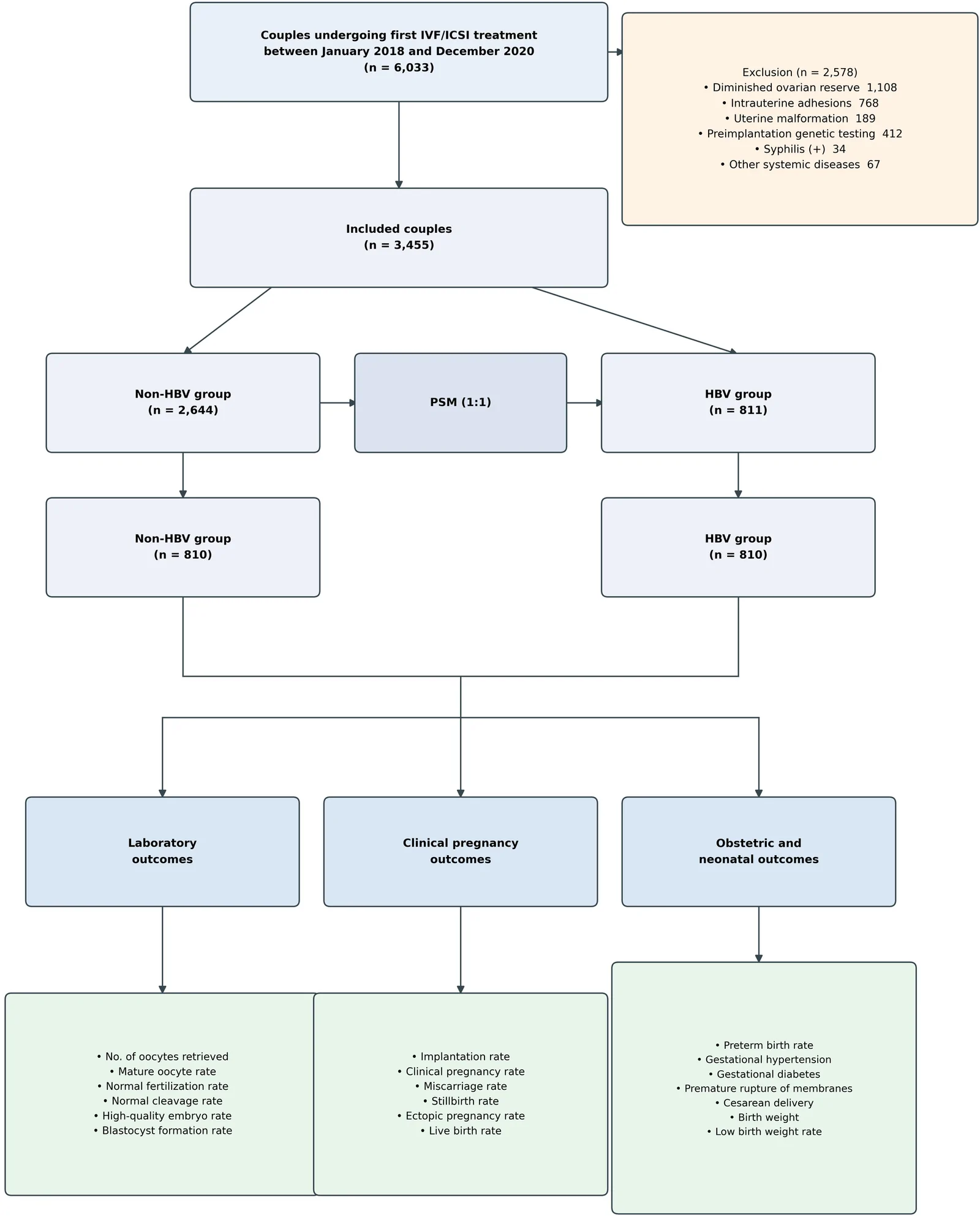
Flow chart of the study.

This retrospective study screened and analyzed data from 3,455 cycles involving couples with infertility who underwent IVF/ICSI treatment for the first time at the Reproductive Medicine Center of Fujian Provincial Maternal and Child Health Hospital from January 2018 to December 2020. Women who were seropositive for hepatitis B surface antigen (HBsAg), hepatitis B core antibody (anti-HBc), and either hepatitis B e antigen (HBeAg) or hepatitis B e antibody (anti-HBe) were classified into the HBV group, provided that their male partners were HBsAg-negative (*n* = 811 cycles). Couples in whom both partners were HBsAg-negative (2,644 cycles) formed the non-HBV control group. The inclusion criteria were having received IVF/ICSI for the first time and having undergone a fresh embryo transfer cycle. Cycles that were cancelled before oocyte retrieval or embryo transfer, that adopted a freeze-all strategy, or that yielded no transferable embryos were not part of the source screening population. The exclusion criteria were diminished ovarian reserve [diagnosed according to the Bologna criteria (16)], a history of intrauterine adhesions, uterine malformations, or other known gynecological diseases, chromosomal abnormalities in either partner identified by preimplantation genetic testing, presence of other systemic diseases such as pituitary tumor and Cushing's syndrome, and either partner being HIV-positive or positive for *Treponema pallidum* infection (Figure 1). All couples gave written informed consent for IVF/ICSI-assisted conception treatment. This study adhered to the Declaration of Helsinki and was approved by the Ethics Review Committee of Fujian Maternity and Child Health Hospital (approval number: 2023KYLLR01081) and registered with the Chinese Clinical Trial Registry (ChiCTR2100053492).

### 2.2 Ovarian stimulation

Ovarian stimulation was performed using a long-acting gonadotropin-releasing hormone agonist regimen or a gonadotropin-releasing hormone antagonist regimen. Bilateral ovarian antral follicle size and number were monitored using ultrasound on days 2–5 of the menstrual cycle, and the regimen was determined by the physician based on the patient's basal endocrine levels, anti-Müllerian hormone levels, age, and body mass index (BMI). Exogenous gonadotropins, such as recombinant human follicle stimulating hormone, were injected to promote follicle growth until triggering was administered when at least two follicles were ≥18 mm in diameter or >50% were ≥16 mm in diameter. Oocytes were retrieved 36 h later via transvaginal ultrasound guidance.

### 2.3 *In vitro* fertilization and embryo culture

After oocyte retrieval, conventional IVF or ICSI was performed, depending on sperm quality, followed by embryo morphological evaluation. Good-quality cleavage embryos were scored as previously described (17). Blastocyst scoring was based on the Gardner and Schoolcraft system (18). Oocyte maturity was assessed at the time of denudation/insemination, and oocytes were classified as grade II, defined as mature oocytes that had extruded the first polar body, or grade I, defined as immature oocytes; the oocyte maturation rate was calculated as the number of grade II oocytes divided by the total number of oocytes retrieved. The normal fertilization rate was calculated as the number of normally fertilized oocytes, identified by the presence of two pronuclei, divided by the total number of oocytes retrieved, rather than by the number of mature oocytes. High-quality embryos were selected for transfer and a maximum of two embryos were transferred per cycle.

### 2.4 Fresh embryo transfer and luteal support

Depending on the patient's condition and according to our center's routine, 1–2 embryos with optimal cleavage-stage morphology or blastocyst scores were selected for transfer using abdominal ultrasound guidance. Cleavage-stage embryos were transferred three days after oocyte retrieval, while blastocyst-stage embryos were transferred five or six days after oocyte retrieval. Any embryos that were not transferred were cryopreserved by vitrification with the couple's informed consent. Post-transfer luteal phase support was provided using progesterone preparations.

### 2.5 Determination of pregnancy outcomes

Serum HCG was measured 14 days after embryo transfer, with values of ≥10 mIU/mL indicating biochemical pregnancy. Clinical pregnancy was defined as the visualization of at least one gestational sac with fetal heartbeat on transvaginal ultrasound performed approximately 30 days after embryo transfer (19). For the purposes of this study, miscarriage was defined as pregnancy loss before 20 completed weeks of gestation, whereas stillbirth was defined as fetal death at or after 20 completed weeks of gestation, based on contemporary ACOG guidance (20, 21). Live birth was defined as the delivery of at least one live neonate (22). Preterm birth was defined as delivery before 37 completed weeks of gestation.

### 2.6 Statistical analyses

SPSS version 26.0 (IBM Corp. Armonk, NY, USA) was used for data analyses. The Shapiro–Wilk test was used to determine whether continuous variables were normally distributed before the two groups were compared. Normally distributed continuous variables are presented as mean ± standard deviation, and differences between groups were compared using the independent-samples t-test. Non-normally distributed continuous variables are presented as medians (interquartile ranges), and differences between groups were compared using the Mann–Whitney U test. Categorical variables are presented as percentages (n/N), and differences between groups were compared using the chi-square test or Fisher's exact test, as appropriate. *P* < 0.05 (two-sided) was considered statistically significant. For binary outcomes, odds ratios (OR) and 95% confidence intervals were additionally estimated using logistic regression. When a woman could contribute more than one observation to an outcome, a cluster-robust (sandwich) variance estimator was used, with the individual woman as the clustering unit before matching and the matched pair as the clustering unit after matching. For all other outcomes, standard logistic regression was used.

Propensity score matching (PSM) was used to adjust for imbalances in baseline characteristics between the HBV and non-HBV groups. Propensity scores were estimated using a logistic regression model that included the following baseline covariates: female age, male age, body mass index, duration of infertility, basal follicle-stimulating hormone, luteinizing hormone, and anti-Müllerian hormone levels, basal antral follicle count (AFC), ovarian stimulation protocol, and insemination method (IVF/ICSI). Missing AFC values were imputed using the group median, with a missing-value indicator included as an additional covariate. Hormone levels on the day of hCG trigger and the number or developmental stage of transferred embryos were not included in the propensity score model, as these may represent mediators rather than baseline confounders. The HBV and non-HBV groups were matched at 1:1 using nearest-neighbor matching without replacement (random order, caliper width: 0.05 on the logit scale, approximately 0.2 standard deviations of the logit-transformed propensity score). Covariate balance after matching was assessed using standardized mean differences (SMD), with SMD < 0.1 considered indicative of adequate balance. The overall distribution of propensity scores between groups was additionally compared using the Kolmogorov–Smirnov test and an independent-samples t-test.

## 3 Results

### 3.1 Baseline characteristics

**Figure 2:**
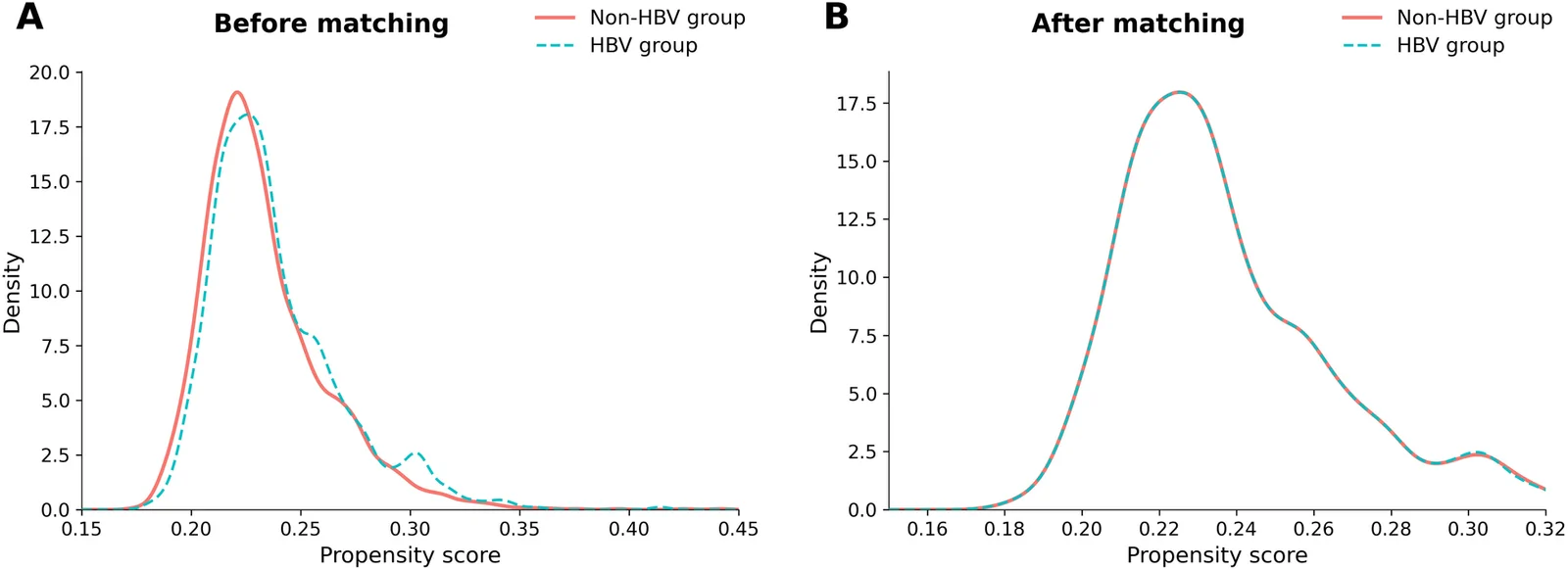
Propensity score density distributions before **(A)** and after **(B)** matching. The non-HBV group is shown in red and the HBV group in green. After matching, the two distributions show substantial overlap, indicating adequate covariate balance.

Among 6,033 IVF/ICSI patients screened, 3,455 cycles were enrolled, including 811 HBV-infected women and 2,644 HBV-negative controls (Figure 1). Before matching, basal LH levels, ovarian stimulation protocol, E^2^ and progesterone levels on hCG day, and insemination method (IVF/ICSI) differed significantly between the two groups (*P* < 0.05, Table 1). Propensity-score matching yielded 810 well-matched pairs. Standardized mean differences for all covariates included in the matching model were <0.1 after matching (Table 1); hCG-day E^2^ remained modestly higher in the HBV group after matching (*P* = 0.001). The propensity score distribution plot confirmed substantial overlap between the matched groups (Figure 2), with no significant difference between groups in the overall distributions (*P* = 1.000; *P* = 0.991).

**Table 1:** Baseline characteristics of participants before and after propensity score matching.

Variable	Before matching				After matching			
	Control (*n* = 2644)	HBV (*n* = 811)	*P*-value	SMD	Control (*n* = 810)	HBV (*n* = 810)	*P*-value	SMD
Age, y								
Male	33.00 (30.00, 37.00)	34.00 (30.50, 37.00)	0.769	0.012	33.00 (30.00, 37.00)	34.00 (30.25, 37.00)	0.535	0.018
Female	32.00 (29.00, 35.00)	32.00 (29.00, 35.00)	0.991	0.000	31.00 (29.00, 35.00)	32.00 (29.00, 35.00)	0.622	0.018
BMI, kg/m^2^	21.36 (19.63, 23.44)	21.48 (19.70, 23.44)	0.290	0.042	21.41 (19.73, 23.52)	21.49 (19.72, 23.44)	0.843	−0.006
FSH, IU/L	6.29 (5.30, 7.48)	6.28 (5.31, 7.46)	0.867	0.021	6.24 (5.26, 7.69)	6.28 (5.31, 7.46)	0.842	−0.014
LH, IU/L	3.30 (2.40, 4.40)	3.50 (2.55, 4.70)	0.010*	0.088	3.40 (2.50, 4.50)	3.50 (2.52, 4.67)	0.358	0.019
AMH, ng/mL	2.78 (1.68, 4.59)	3.01 (1.76, 4.90)	0.300	0.040	2.79 (1.70, 4.49)	3.00 (1.79, 4.89)	0.156	0.027
AFC	13.00 (9.00, 17.00)	13.00 (9.00, 18.00)	0.407	0.029	12.00 (9.00, 17.00)	13.00 (9.00, 18.00)	0.720	0.013
Infertility duration, y	3.00 (2.00, 5.00)	3.00 (2.00, 5.00)	0.138	0.060	3.00 (2.00, 5.00)	3.00 (2.00, 5.00)	0.391	−0.055
Infertility type, %			0.234	−0.049			0.025*	−0.111
Primary	1278 (48.3)	372 (45.9)			416 (51.4)	371 (45.8)		
Secondary	1366 (51.7)	439 (54.1)			394 (48.6)	439 (54.2)		
Infertility causes, %			0.648				0.803	
Tubal factor	43.68 (1155/2644)	45.01 (365/811)		0.027	43.58 (353/810)	44.57 (361/810)		0.020
Male factor	28.37 (750/2644)	28.98 (235/811)		0.013	27.78 (225/810)	28.77 (233/810)		0.022
Unexplained	15.73 (416/2644)	13.93 (113/811)		−0.051	14.44 (117/810)	13.95 (113/810)		−0.014
Others	12.22 (323/2644)	12.08 (98/811)		−0.004	13.58 (110/810)	12.10 (98/810)		−0.044
COH protocol, %			<0.001*				0.741	
GnRH-agonist	56.88 (1504/2644)	70.78 (574/811)		0.023	69.51 (563/810)	70.86 (574/810)		0.030
GnRH-antagonist	29.99 (793/2644)	26.88 (218/811)		−0.024	28.52 (231/810)	26.91 (218/810)		−0.036
Others^a^	13.13 (347/2644)	2.34 (19/811)		0.002	1.98 (16/810)	2.22 (18/810)		0.017
Gn duration, d	12.00 (10.00, 13.00)	12.00 (10.00, 13.00)	0.589	−0.022	12.00 (10.00, 13.00)	12.00 (10.00, 13.00)	0.475	−0.023
Gn dosage, IU	2550.00 (2025.00, 3150.00)	2475.00 (2025.00, 3150.00)	0.487	−0.028	2550.00 (2025.00, 3150.00)	2475.00 (2025.00, 3150.00)	0.502	−0.030
E^2^ on HCG day, pg/mL	2199.50 (1310.00, 3252.50)	2352.00 (1431.00, 3604.00)	<0.001*	0.151	2207.00 (1311.00, 3080.25)	2356.00 (1433.00, 3605.00)	0.001*	0.180
*P* on HCG day, ng/mL	0.60 (0.40, 0.90)	0.70 (0.40, 1.00)	0.007*	0.107	0.60 (0.40, 0.90)	0.70 (0.40, 1.00)	0.009*	0.132
Insemination method, %			<0.001*	−0.034			0.858	0.009
IVF	70.23 (1857/2644)	77.31 (627/811)			77.78 (630/810)	77.41 (627/810)		
ICSI	29.77 (787/2644)	22.69 (184/811)			22.22 (180/810)	22.59 (183/810)		
No. of embryos transferred	1.78 ± 0.42	1.79 ± 0.41	0.357	0.037	1.79 ± 0.41	1.79 ± 0.41	0.903	0.006
Embryo stage transferred, %			0.375	0.044			0.753	0.018
Cleavage embryo	95.76 (2532/2644)	96.55 (783/811)			93.21 (755/810)	93.58 (758/810)		
Blastocyst	4.24 (112/2644)	3.45 (28/811)			6.17 (50/810)	5.80 (47/810)		

Continuous variables are presented as the median (interquartile range) or mean ± standard deviation. Categorical variables are presented as % (n/N). *P < 0.05 was considered statistically significant.

BMI, body mass index; FSH, follicle-stimulating hormone; LH, luteinizing hormone; AMH, anti-Müllerian hormone; AFC, antral follicle count; COH, controlled ovarian hyperstimulation; GnRH, gonadotropin-releasing hormone; Gn, gonadotropin; HCG, human chorionic gonadotropin; E^2^, estradiol; P, progesterone; IVF, *in vitro* fertilization; ICSI, intracytoplasmic sperm injection.

a Others (COH protocol): including mild stimulation and luteal phase stimulation protocols.

### 3.2 Laboratory outcomes

Before PSM, the number of oocytes retrieved differed significantly between the HBV and non-HBV groups (*P* = 0.028), but this difference was not significant after matching (*P* = 0.091) (Table 2). Downstream laboratory indicators showed no significant differences between the two groups based on cluster-robust logistic regression, both before and after matching, with all odds ratios close to 1 and 95% confidence intervals spanning the null (Table 2). In a *post hoc* analysis stratified by insemination method, the oocyte maturation rate was modestly higher in the HBV group among ICSI cycles (55.56% vs. 51.98%, *P* = 0.040) but not among IVF cycles.

**Table 2:** Laboratory outcomes before and after PSM.

Variable	Before PSM				After PSM			
	Control (*n* = 2644)	HBV (*n* = 811)	*P*-value	OR (95% CI)	Control (*n* = 810)	HBV (*n* = 810)	*P*-value	OR (95% CI)
No. of oocytes retrieved	8.00 (5.00, 12.00)	9.00 (6.00, 12.00)	**0**.028*	–	8.00 (5.00, 12.00)	9.00 (6.00, 12.00)	0.091	–
Mature oocyte rate, %^a^	52.70 (12207/23165)	52.85 (3946/7467)	0.893	1.006 (0.922–1.097)	52.38 (3718/7098)	52.84 (3945/7466)	0.749	1.019 (0.910–1.140)
Normal fertilization rate, %^a^	82.07 (19011/23165)	81.73 (6103/7467)	0.673	0.977 (0.878–1.087)	82.01 (5821/7098)	81.73 (6102/7466)	0.772	0.981 (0.860–1.119)
Normal cleavage rate, %^a^	93.85 (17842/19011)	93.53 (5708/6103)	0.458	0.947 (0.819–1.094)	93.87 (5464/5821)	93.53 (5707/6102)	0.558	0.946 (0.786–1.139)
High-quality embryo rate, %^a^	59.34 (10587/17842)	57.83 (3301/5708)	0.157	0.940 (0.862–1.024)	58.66 (3205/5464)	57.82 (3300/5707)	0.535	0.966 (0.867–1.077)
Blastocyst formation rate, %^a^	63.82 (4753/7448)	63.49 (1501/2364)	0.843	0.987 (0.870–1.120)	64.45 (1445/2242)	63.49 (1501/2364)	0.594	0.959 (0.823–1.118)

Values are presented as % (n/N) or median (interquartile range). *P < 0.05 was considered statistically significant.

a Cluster-robust (sandwich) variance estimator used to account for multiple oocytes or embryos contributed by each woman. OR (95% CI) is not applicable to the number of oocytes retrieved, a continuous variable (*P*-value from Mann–Whitney *U* test).

### 3.3 Clinical pregnancy and delivery outcomes

Before PSM, no significant differences were found between the HBV and non-HBV groups in implantation rate, clinical pregnancy rate, multiple pregnancy rate, ectopic pregnancy rate, or live birth rate (all *P* > 0.05, Table 3). Neither the miscarriage rate nor the stillbirth rate differed significantly between groups (both *P* > 0.05, Table 3).

**Table 3:** Clinical pregnancy and delivery outcomes before and after PSM.

Variable	Before PSM				After PSM			
	Control	HBV	*P*-value	OR (95% CI)	Control	HBV	*P*-value	OR (95% CI)
Implantation rate, %^a^^,^^e^	33.85 (1590/4697)	30.97 (450/1453)	0.775	1.023 (0.874–1.197)	32.76 (475/1450)	30.99 (450/1452)	0.307	0.960 (0.790–1.167)
Clinical pregnancy rate, %^b^	46.94 (1241/2644)	44.39 (360/811)	0.203	0.902 (0.770–1.057)	45.43 (368/810)	44.44 (360/810)	0.690	0.961 (0.790–1.169)
Multiple pregnancy rate, %^c^	31.35 (389/1241)	29.17 (105/360)	0.431	0.902 (0.697–1.166)	32.61 (120/368)	29.17 (105/360)	0.315	0.851 (0.621–1.166)
Miscarriage rate, %^c^	10.07 (125/1241)	12.78 (46/360)	0.144	1.308 (0.912–1.876)	9.24 (34/368)	12.78 (46/360)	0.127	1.439 (0.900–2.301)
Stillbirth rate, %^c^	0.89 (11/1241)	1.39 (5/360)	0.403	1.575 (0.544–4.563)	1.09 (4/368)	1.39 (5/360)	0.712	1.282 (0.341–4.812)
Ectopic pregnancy rate, %^c^	2.66 (33/1241)	3.61 (13/360)	0.343	1.371 (0.714–2.634)	3.53 (13/368)	3.61 (13/360)	0.955	1.023 (0.468–2.238)
Live birth rate, %^b^	34.80 (920/2644)	32.55 (264/811)	0.239	0.904 (0.765–1.069)	32.96 (267/810)	32.59 (264/810)	0.874	0.983 (0.799–1.210)
Preterm birth rate, %^d^	11.10 (103/928)	15.73 (42/267)	**0**.042*	1.495 (1.014–2.204)	7.78 (21/270)	15.73 (42/267)	**0**.004*	2.213 (1.272–3.852)

Values are presented as % (n/N). Bold values indicate statistically significant differences (*P* < 0.05). *P < 0.05 was considered statistically significant.

ᵃ Denominator: total number of embryos transferred.

ᵇ Denominator: total number of cycles (before PSM: Control *n* = 2644, HBV *n* = 811; after PSM: both *n* = 810).

ᶜ Denominator: number of clinical pregnancies (before PSM: Control *n* = 1241, HBV *n* = 360; after PSM: Control *n* = 368, HBV *n* = 360).

ᵈ Denominator: number of live births (before PSM: Control *n* = 928, HBV *n* = 267; after PSM: Control *n* = 270, HBV *n* = 267).

e Cluster-robust (sandwich) variance estimator used to account for multiple embryos transferred per woman.

### 3.4 Obstetric and neonatal outcomes

Among women who achieved a live birth after fresh embryo transfer, 928 deliveries occurred in the non-HBV group and 267 in the HBV group before matching; after matching, 270 and 267 deliveries were available for analysis in the non-HBV and HBV groups, respectively. Rates of gestational hypertension, gestational diabetes, premature rupture of membranes, and cesarean delivery did not differ significantly between the two groups either before or after PSM (all *P* > 0.05, Table 4). Similarly, neonatal sex ratio, mean birth weight, and low birth weight rate were comparable between groups (all *P* > 0.05, Table 4). However, the preterm birth rate remained significantly higher in the HBV group than in the non-HBV group both before PSM (*P* = 0.041) and after PSM (*P* = 0.004) (Table 3). When stratified by singleton and multiple pregnancy, this difference remained significant in both subgroups after matching (singleton: 6.57% vs. 2.15%, *P* = 0.036; twin: 42.03% vs. 20.24%, *P* = 0.003). A Cochran-Mantel-Haenszel analysis stratified by pregnancy plurality yielded a statistically significant association (adjusted OR 2.960, *P* < 0.001).

**Table 4:** Obstetric and neonatal outcomes before and after PSM.

Variable	Before PSM				After PSM			
	Control (*n* = 928)	HBV (*n* = 267)	*P*-value	OR (95% CI)	Control (*n* = 270)	HBV (*n* = 267)	*P*-value	OR (95% CI)
Obstetric outcomes								
Gestational hypertension, %	3.99 (37/928)	4.49 (12/267)	0.713	1.133 (0.582–2.205)	4.44 (12/270)	4.49 (12/267)	0.978	1.012 (0.446–2.294)
Gestational diabetes, %	16.92 (157/928)	18.73 (50/267)	0.491	1.132 (0.796–1.609)	18.15 (49/270)	18.35 (49/267)	0.951	1.014 (0.654–1.571)
PROM, %	27.91 (259/928)	26.97 (72/267)	0.762	0.954 (0.702–1.295)	28.15 (76/270)	26.59 (71/267)	0.686	0.925 (0.633–1.352)
Cesarean delivery, %	64.76 (601/928)	60.67 (162/267)	0.220	0.839 (0.634–1.111)	67.78 (183/270)	60.67 (162/267)	0.086	0.733 (0.515–1.045)
Neonatal outcomes^a^								
Sex, %^b^			0.773	0.965 (0.759–1.227)			0.727	0.948 (0.701–1.281)
Male, %	52.38 (626/1195)	51.64 (173/335)			52.82 (187/354)	51.64 (173/335)		
Female, %	47.62 (569/1195)	48.36 (162/335)			47.18 (167/354)	48.36 (162/335)		
Birth weight, kg	2.90 (2.50, 3.30)	2.92 (2.50, 3.33)	0.335	–	2.90 (2.55, 3.27)	2.92 (2.50, 3.33)	0.776	–
Low birth weight, %^b^	24.10 (288/1195)	23.58 (79/335)	0.873	0.973 (0.695–1.362)	20.62 (73/354)	23.58 (79/335)	0.349	1.189 (0.786–1.796)

Values are presented as % (n/N) or median (interquartile range). *P* < 0.05 was considered statistically significant.

PROM, premature rupture of membranes. Cesarean delivery: original text used “cesarean section”; updated to preferred BMC terminology.

a Neonatal counts (unmatched: *n* = 1,195 and 335; matched: *n* = 354 and 335) exceed delivery counts due to multiple pregnancies.

Birth weight is presented as median (interquartile range, kg). Low birth weight is defined as <2,500 g. OR (95% CI) is not applicable to birth weight, a continuous variable; the group comparison for this variable is based on the Mann–Whitney *U* test shown in the *P*-value column.

b Cluster-robust (sandwich) variance estimator used to account for multiple neonates contributed by each woman in multiple pregnancies.

## 4 Discussion

In this propensity score-matched cohort study of women undergoing fresh IVF/ICSI embryo transfer, maternal HBV infection was not associated with significant differences in laboratory outcomes, clinical pregnancy outcomes, or most obstetric and neonatal outcomes. A higher preterm birth rate was observed in the HBV group and remained evident after adjustment for pregnancy plurality. However, given the multiple outcomes examined and the potential for residual unmeasured confounding, this finding should be interpreted cautiously and considered hypothesis-generating rather than definitive.

Our findings on clinical pregnancy and live birth rates are consistent with the large cohort by Wang et al., who reported no impact of maternal chronic HBV infection on pregnancy outcomes in infertile patients undergoing their first IVF cycle (12), as well as with the meta-analysis by Farsimadan et al. and the review by Mak and Lao (15, 23). They contrast with the small case-control study by Pirwany et al., who reported markedly lower implantation and pregnancy rates in HBV-positive couples (24), and with Lam et al., who reported the opposite—markedly higher rates in HBV carriers (14). Both of these earlier studies were limited by sample sizes of fewer than 300 couples and did not control for the substantial baseline imbalances we observed in our unmatched cohort, including differences in basal LH, ovarian stimulation protocol, hormonal response, and insemination method. After PSM adjusted for these confounders, no significant differences in laboratory or clinical outcomes were observed, supporting the view that previously reported associations may have been driven by unmeasured baseline differences rather than by HBV infection itself.

Although HBV DNA has been found integrated in germ cell genomes and HBV DNA has also been detected in follicular fluid (4–6), raising theoretical concerns about impaired gametogenesis and early embryo development, our results did not show any measurable reduction in IVF/ICSI success rates among HBV-infected women. This is in line with the reports by Wang et al. (12) and the meta-analysis by Farsimadan et al. (15). It is possible that routine laboratory procedures in IVF/ICSI, such as oocyte washing and the ICSI process itself, effectively reduce viral exposure at the gamete level. Moreover, most HBV-infected women presenting for fertility treatment are chronic carriers with low viral loads, which may be insufficient to cause direct damage to oocytes or embryos. That said, accumulating evidence indicates female HBV infection is associated with reduced cumulative pregnancy rates in patients undergoing both fresh and frozen-thawed IVF cycles (13), so the issue remains open, particularly when outcomes beyond a single fresh transfer are considered. The modestly higher oocyte maturation rate observed in the HBV group within ICSI cycles should likewise be interpreted with caution, given the number of comparisons performed in this exploratory, stratified analysis.

The preterm birth rate in our HBV group was notably higher than in controls, a finding that warrants attention. This is not an isolated observation: Xu et al. reported a similar trend in a large cohort of naturally conceived pregnancies among Chinese women (25), and a meta-analysis of 35 cohort studies by Afraie et al. also confirmed a positive association between maternal HBV infection and preterm delivery (26). Taken together, these data suggest that the relationship between chronic HBV infection and preterm birth is not unique to ART populations.

The underlying mechanisms remain incompletely understood, but several plausible pathways have been proposed. Chronic HBV carriage is known to maintain a low-grade inflammatory state with elevated circulating levels of TNF-α, IL-6, and IL-8 (10), all of which have been implicated in premature uterine contractions, membrane rupture, and cervical remodeling. In addition, HBV has been shown to infect placental trophoblasts and villous stromal cells through the maternal circulation, potentially triggering chorioamnionitis or deciduitis and thereby compromising placental integrity (10, 25). Oxidative stress induced by placental HBV infection may further damage vascular endothelial function at the feto-maternal interface, predisposing to preterm delivery (27). It should be noted, however, that not all published data point in the same direction. Lin et al. found no significant association between maternal HBV status and gestational age in an IVF-ET cohort (28), a discrepancy that may reflect differences in viral load profiles, antiviral treatment practices, or population characteristics across studies.

The strengths of this study include a relatively large sample size from a high-prevalence region, the use of propensity score matching with rigorous covariate balance to minimize confounding, and the comprehensive reporting of outcomes spanning laboratory, clinical pregnancy, obstetric, and neonatal endpoints within a single cohort. Several limitations should also be acknowledged. First, as a retrospective single-center study, residual confounding cannot be excluded despite PSM. Second, and most importantly, detailed virological, hepatic, and pregnancy-related clinical data—including HBV-DNA viral load, liver function, antiviral treatment, disease severity, relevant co-infections, family history of preterm delivery, and HBV-related pregnancy complications—were not systematically available in this retrospective cohort. In addition, detailed information on infection-control or maternal-fetal management practices and prenatal care was unavailable. These limitations precluded stratified analyses by viral activity or disease severity and limited our ability to fully characterize the clinical factors underlying the observed preterm-birth association. Third, the non-HBV control group was defined by HBsAg negativity alone; anti-HBc and anti-HBs results were not systematically available, so women with resolved or occult HBV infection could not be excluded from this group, which may have attenuated rather than exaggerated the observed differences between groups. Fourth, we restricted our analysis to fresh embryo transfer cycles and did not assess cumulative live birth rates or long-term outcomes from frozen-thawed embryo transfers, which may limit generalizability; as with any retrospective analysis, selection bias cannot be entirely excluded. Fifth, a portion of follow-up data on obstetric and neonatal outcomes was collected through telephone interviews, which may introduce recall or reporting bias. Sixth, the non-significant difference in miscarriage rate should be interpreted with caution. The relatively limited number of miscarriage events may have reduced the statistical precision of this comparison, and an exploratory *post hoc* power calculation yielded only 33.2% power to detect the observed 3.5-percentage-point difference (12.78% vs. 9.24%) at a two-sided *α* level of 0.05. Future prospective studies that incorporate detailed virologic and treatment data, and that follow patients through cumulative cycles and long-term offspring outcomes, are needed to fully clarify the impact of maternal HBV infection on ART. Importantly, the association between HBV infection and preterm birth in our study was observed as a crude comparison, after propensity-score-matched adjustment, and remained robust in a Cochran-Mantel-Haenszel analysis adjusting for singleton/multiple pregnancy status. However, this was one of 21 outcome comparisons reported in this study, and the association did not remain statistically significant after Benjamini-Hochberg correction for multiple testing. In light of this, the potential for residual confounding, and the other limitations discussed above, this finding should be regarded as hypothesis-generating rather than definitive.

## 5 Conclusions

In this propensity score-matched cohort of women undergoing fresh IVF/ICSI embryo transfer, maternal HBV infection was not associated with significant differences in laboratory outcomes, clinical pregnancy outcomes, or most obstetric and neonatal outcomes. A higher preterm birth rate was observed among women with HBV infection and remained evident after adjustment for pregnancy plurality; however, this finding did not remain statistically significant after accounting for multiple comparisons and may be influenced by residual unmeasured confounding. Therefore, the observed association with preterm birth should be considered hypothesis-generating rather than definitive. Further prospective, multicenter studies incorporating detailed virological, hepatic, and obstetric data are warranted to validate these findings and clarify the potential mechanisms underlying preterm birth in pregnancies affected by maternal HBV infection.

## Data Availability

The raw data supporting the conclusions of this article will be made available by the authors, without undue reservation.
